# Network meta-analysis of different preoperative hormone therapies for improving postoperative complications in pediatric hypospadias

**DOI:** 10.3389/fendo.2026.1842765

**Published:** 2026-06-24

**Authors:** Ling Wu, Wenmei Wang, Qi Zhao, Xing Liu, Yun Pan, Bowen Yang, Quanqing Xiong, Hongqiang Deng, Jiabo Chen

**Affiliations:** 1 Department of Pediatric Surgery, Guangxi Academy of Medical Sciences, The People's Hospital of Guangxi Zhuang Autonomous Region, Nanning, Guangxi, China; 2 Department of Surgery, Guangxi Zhuang Autonomous Region Maternal and Child Health Care Hospital, Nanning, Guangxi, China

**Keywords:** androgen, hormone therapy, hypospadias, network meta-analysis, postoperative complications

## Abstract

**Objective:**

To evaluate and compare the efficacy of different preoperative hormonal therapies for preventing postoperative complications after hypospadias repair in pediatric patients through a systematic review and network meta-analysis.

**Methods:**

PubMed, Web of Science, Cochrane Library, and CNKI were systematically searched (inception to March 15, 2026). Randomized controlled trials (RCTs) enrolling pediatric patients with hypospadias who received preoperative hormone therapy were included. Risk of bias was assessed using the Cochrane RoB 2 tool. The primary outcomes were postoperative urethrocutaneous fistula, glans dehiscence, and meatal stenosis. Bayesian network meta-analysis was performed using the gemtc package in R 4.3.1, and SUCRA was used to rank intervention efficacy. Sensitivity analysis excluding non-distal cohorts was performed to assess robustness.

**Results:**

Six RCTs (715 patients) were included. Direct pairwise meta-analysis showed that androgen therapy reduced the odds of urethrocutaneous fistula (pooled OR = 0.474; 95% CI: 0.241–0.934; P = 0.031). In the sensitivity analysis restricted to distal hypospadias only, the protective trend persisted but did not reach statistical significance (OR = 0.613; 95% CI: 0.285–1.319). No significant benefit of androgen was observed for glans dehiscence or meatal stenosis. Estrogen did not significantly reduce any complication. SUCRA analysis identified androgen as the most probable effective intervention for fistula prevention.

**Conclusion:**

Preoperative androgen therapy may reduce the odds of postoperative urethrocutaneous fistula in pediatric patients undergoing hypospadias repair, though this benefit was primarily observed in mixed-severity cohorts. No significant protective effect was demonstrated for glans dehiscence or meatal stenosis. Further high-quality RCTs with standardized protocols and long-term follow-up are warranted, particularly in distal hypospadias populations.

**Systematic Review Registration:**

https://www.crd.york.ac.uk/PROSPERO/view/CRD420261351057, identifier CRD420261351057.

## 1 Introduction

Hypospadias is one of the most common congenital malformations of the male external genitalia, characterized by a ventrally placed urethral meatus rather than at the normal tip of the glans. According to the position of the urethral meatus, it is classified into distal (glanular/coronal, accounting for 50%-70%), midshaft, and proximal (penoscrotal/perineal) types (1). Epidemiological data show that the pooled global prevalence of hypospadias is approximately 20.9 per 10,000 live births (95% confidence interval [CI]: 19.2–22.6/10,000). Based on joinpoint regression analysis of big data from 27 surveillance programs of the International Clearinghouse for Birth Defects Surveillance and Research (ICBDSR) over 31 years (1980–2010), the global total prevalence showed a significant upward trend during the study period, increasing to 1.6 times the baseline level, with an average annual increase of approximately 0.25 per 10,000 (P < 0.05) (2). If not repaired in a timely and appropriate manner, hypospadias can affect the child’s voiding function, sexual health, and long-term psychosocial adaptation (3).

Currently, surgical repair is the main treatment for hypospadias; however, the success rate is constrained by multiple factors, including the position of the urethral meatus, the degree of penile development, vascularization of the prepuce, and surgeon experience (4, 5). Clinically, a considerable proportion of children present with penile hypoplasia or insufficient glans width, a phenomenon related to younger age at surgery and the co-occurrence of disorders of sex development (DSD) in some children, the latter often caused by genetic or chromosomal abnormalities (6). These anatomical characteristics increase the difficulty of intraoperative exposure and reconstruction, significantly increasing the technical challenge of restoring the urethral meatus to its normal anatomical position. To improve the operative field and increase glans width, preoperative hormone therapy (PHT) has become an important adjunctive strategy. Since Immergut et al. (1971) first reported that testosterone could promote penile growth (7), subsequent studies have gradually expanded the options for PHT, including human chorionic gonadotropin (hCG) (8) and, more recently, estrogen preparations that have been attempted (9).

However, the clinical benefits of PHT are inconsistent in existing evidence and vary by complication type. The core pathological mechanism of urethrocutaneous fistula is local ischemia at the suture line of the urethroplasty; androgen can directly correct this hemodynamic deficit by upregulating the vascular endothelial growth factor (VEGF)-dependent neovascularization pathway (10, 11). In contrast, glans dehiscence is essentially a mechanical separation at the surgical suture, mainly governed by suture tension, urethral plate quality, and surgical technique (12, 13); meatal stenosis is primarily caused by scar contracture and reconstruction technique (14). It is noteworthy that although PHT can increase glans width, glans size is only an auxiliary rather than a determinant factor for dehiscence; suture tension and technical factors are the dominant variables. Therefore, the benefit of PHT is most clearly established for vascular-dependent complications (fistula) but limited for mechanical/technique-dependent complications (glans dehiscence, urethral stenosis). In addition, different complications have characteristic time windows of clinical manifestation: glans dehiscence mostly appears within 2 months postoperatively, whereas urethrocutaneous fistula and meatal stenosis are mostly diagnosed within 6 months postoperatively (15), with a minority presenting later. This temporal distribution difference requires that an adequate follow-up duration be established in efficacy assessments to cover the typical occurrence windows of all complication types.

Although preoperative hormone therapy has been widely used to optimize surgical conditions for hypospadias repair, existing evidence suggests that its preventive effects on postoperative complications vary by complication type. Previous studies have mostly been direct pairwise comparisons of a single hormone versus control, and systematic network evidence for the relative efficacy of different hormone regimens is lacking. This study systematically compared the effects of different PHT regimens on postoperative complications in pediatric patients with hypospadias through network meta-analysis (NMA) to provide evidence-based support for clinical decision-making.

## 2 Materials and methods

This systematic review and network meta-analysis were conducted in accordance with the Preferred Reporting Items for Systematic Reviews and Meta-Analyses (PRISMA) 2020 guidelines (16) and registered with the international prospective register of systematic reviews PROSPERO (registration number: CRD420261351057).

### 2.1 Literature search strategy

The following electronic databases were systematically searched: PubMed (MEDLINE, U.S. National Library of Medicine), Web of Science Core Collection (Clarivate Analytics, Philadelphia, PA, USA), Cochrane Library (Cochrane Collaboration, Oxford, UK), and China National Knowledge Infrastructure (CNKI; Tongfang Knowledge Network, Beijing, China), from the inception of each database to March 15, 2026. The search strategy combined Medical Subject Headings (MeSH) terms with free-text words, structured by the PICOS framework. Language was restricted to English and Chinese. All search results were imported into EndNote X9 (Clarivate Analytics, Philadelphia, PA, USA; version X9.3.3) for deduplication management.

PubMed search strategy: (((“hypospadias”[MeSH Terms]) OR (“hypospadias”[Title/Abstract]) OR (“urethral malposition”[Title/Abstract]) OR (“ventral urethral meatus”[Title/Abstract])) AND ((“preoperative hormone therapy”[Title/Abstract]) OR (“preoperative hormonal stimulation”[Title/Abstract]) OR (“testosterone”[Title/Abstract]) OR (“androgen”[Title/Abstract]) OR (“estrogen”[Title/Abstract]) OR (“estradiol”[Title/Abstract]) OR (“human chorionic gonadotropin”[Title/Abstract]) OR (“hCG”[Title/Abstract]) OR (“dihydrotestosterone”[Title/Abstract]) OR (“DHT”[Title/Abstract]))) AND ((“randomized controlled trial”[Publication Type]) OR (“randomized”[Title/Abstract]) OR (“placebo”[Title/Abstract]))).

Filters: English OR Chinese; publication date from inception to March 15, 2026.

Web of Science search strategy: TS=(hypospadias OR “urethral malposition” OR “ventral urethral meatus”) AND TS=(“preoperative hormone therapy” OR “preoperative hormonal stimulation” OR testosterone OR androgen OR estrogen OR estradiol OR “human chorionic gonadotropin” OR hCG OR dihydrotestosterone OR DHT) AND TS=(random* OR trial* OR placebo).

Filters: English OR Chinese; all years to 2026.

Cochrane Library search strategy: (hypospadias OR “urethral malposition”) AND (“preoperative hormone” OR testosterone OR androgen OR estrogen OR hCG OR dihydrotestosterone) in Title, Abstract, OR Keywords.

Filters: Trials; all dates; English OR Chinese.

CNKI search strategy: SU = (“hypospadias”) AND SU = (“preoperative hormone therapy” OR “preoperative androgen therapy” OR “preoperative estrogen therapy” OR “testosterone” OR “estrogen” OR “human chorionic gonadotropin” OR “dihydrotestosterone” OR “androgen”) AND SU = (“randomized” OR “RCT” OR “controlled”).

Filters: all publication years to 2026.

### 2.2 Inclusion and exclusion criteria

Eligibility criteria were formulated according to the PICOS framework:

#### 2.2.1 Inclusion criteria

(1) Study design (S): randomized controlled trials (RCT) published in English or Chinese; (2) Participants (P): male patients aged <18 years with a clinical diagnosis of hypospadias (distal, midshaft, or proximal). Because RCT evidence restricted exclusively to distal hypospadias was limited—only three eligible RCTs with exclusive distal populations were identified—we retained studies with broader severity distributions to ensure a sufficient evidence base for robust network meta-analysis. A pre-planned sensitivity analysis excluding studies that explicitly included proximal or midshaft cohorts (Chen 2015, Pati 2023, Gorduza 2020) was performed to evaluate the impact of disease severity heterogeneity on pooled estimates. (3) Intervention (I): the experimental group received preoperative hormone therapy, including androgen, estrogen, or human chorionic gonadotropin (hCG); the control group received placebo, blank control, or no treatment; (4) Outcomes (O): reporting the incidence of at least one of the following postoperative complications: (i) Urethrocutaneous fistula: defined as an epithelialized abnormal tract between the urethra and the newly reconstructed urethra, manifested as urine leakage from the skin surface during postoperative voiding; (ii) Glans dehiscence: defined as re-separation of the bilateral glans wings after glansplasty, resulting in exposure of the neomeatus or urethral plate; (iii) Meatal stenosis: defined as narrowing of the caliber of the new external urethral meatus due to scar contracture, requiring urethral dilation, calibration, or secondary surgical intervention. The above definitions were confirmed to be substantially consistent across all included studies after cross-checking; (5) Follow-up duration: minimum postoperative follow-up >=3 months to cover the typical occurrence window of urethrocutaneous fistula and meatal stenosis.

#### 2.2.2 Exclusion criteria

(1) Publications not in English or Chinese; (2) Animal studies, case reports, narrative reviews, conference abstracts, letters, commentaries, or expert opinions; (3) Incomplete data from which dichotomous outcome data could not be extracted; (4) Retracted articles; (5) Postoperative follow-up <3 months; (6) Duplicate publications or overlapping patient cohorts (identified by comparing authors, research centers, sample sizes, and time windows; the version with the most complete data or the earliest publication was retained). hCG studies: three potentially eligible RCTs involving hCG were initially identified, but none were included after full-text screening. The reasons for exclusion were as follows: (i) one study did not use randomized grouping and employed a before-after control design, failing to meet the RCT inclusion criterion; (ii) the remaining two studies involved mixed malformation populations (including bladder exstrophy, etc.) and did not report hypospadias subgroup outcomes separately; (iii) none of the hCG-related studies reported the three target complications defined in this study. Therefore, the hCG node was not included in the final network.

### 2.3 Literature screening and data extraction

Two independent reviewers performed screening, independently evaluating titles, abstracts, and full texts at each level. Inter-reviewer agreement was assessed using Cohen’s kappa coefficient, with a requirement of >0.80 before formal screening commenced. Discrepancies were resolved through discussion or consultation with a third reviewer. Data extraction was performed using a pre-designed standardized form that included: first author, publication year, study country, sample size, patient age, hypospadias classification, surgical technique, hormone type, dose, route of administration, treatment duration, follow-up duration, and the number of occurrences of the three complications.

### 2.4 Risk-of-bias assessment

The Cochrane Risk-of-Bias Tool version 2 (RoB 2) (17) was used to assess the methodological quality of included RCTs. The assessment domains included: (1) randomization process, (2) deviations from intended interventions, (3) missing outcome data, (4) measurement of the outcome, and (5) selection of the reported result. Each domain and the overall risk of bias were rated as “low risk,” “some concerns,” or “high risk.” Two reviewers independently performed the ratings, and disagreements were resolved through consensus. Risk-of-bias summary plots were generated using Review Manager software (RevMan version 5.4; Cochrane Collaboration, Copenhagen, Denmark; released 2020).

### 2.5 Statistical analysis

#### 2.5.1 Pairwise meta-analysis

For each outcome, direct pairwise meta-analyses were conducted separately: androgen versus control, and estrogen versus control. Given that all outcomes were dichotomous variables, and all included studies were RCTs, the Mantel-Haenszel method was used to calculate odds ratios (OR) and 95% confidence intervals (CI). Odds ratio was selected as the primary effect measure because it is the standard metric for synthesizing binary outcomes from RCTs, is invariant to the direction of the effect, and provides a symmetric scale on the logit scale that facilitates meta-analysis pooling and heterogeneity assessment. Moreover, OR is the natural effect size for the binomial likelihood model employed in the Bayesian network meta-analysis, ensuring methodological consistency across the direct and indirect comparisons. Heterogeneity was assessed using Cochrane’s Q test and the I² statistic. In addition, sensitivity analyses excluding studies rated as “high risk” or “some concerns” by RoB 2 were performed to examine the impact of risk of bias on the results. Three included studies contained single-zero cells (one arm with zero events). For pairwise meta-analyses with zero-event cells, the Peto odds ratio method was used as it provides valid statistical inference under such conditions. For the Bayesian network meta-analysis, the binomial likelihood specification naturally accommodates zero event counts without requiring continuity correction. The complete 2×2 event tables for all outcomes are provided in **Table 1**.

**Table 1.1:** Complete 2×2 event tables for all outcomes.

Study	Group	Events (n)	Total (N)	Event rate (%)
Gorduza 2020 (26)	Estrogen	17	119	14.29
	Control	19	122	15.57
Pati 2023 (25)	Estrogen	11	29	37.93
	Control	16	31	51.61
Chen 2015 (22)	Androgen	2	34	5.88
	Control	9	36	25.00
Babu 2017 (21)	Androgen	6	94	6.38
	Control	7	92	7.61
Kaya 2007 (23)	Androgen	1	37	2.70
	Control	4	38	10.53
Menon 2017 (24)	Androgen	5	49	10.20
	Control	7	45	15.56

Single-zero cells in Chen 2015 (Androgen: 2 events) and Kaya 2007 (Androgen: 1 event). Peto odds ratio method was applied for zero-event cells in pairwise meta-analyses.

Urethrocutaneous fistula (UF).

**Table 1.2:** Glans dehiscence (GD).

Study	Group	Events (n)	Total (N)	Event rate (%)
Gorduza 2020 (26)	Estrogen	15	114	13.16
	Control	15	116	12.93
Pati 2023 (25)	Estrogen	12	29	41.38
	Control	14	31	45.16
Chen 2015 (22)	Androgen	3	34	8.82
	Control	3	36	8.33
Babu 2017 (21)	Androgen	7	94	7.45
	Control	13	92	14.13
Kaya 2007 (23)	Androgen	0	37	0.00
	Control	3	38	7.89
Menon 2017 (24)	Androgen	7	49	14.29
	Control	0	45	0.00

Single-zero cells in Kaya 2007 (Androgen: 0 events) and Menon 2017 (Control: 0 events). Peto odds ratio method was applied for zero-event cells.

**Table 1.3:** Meatal stenosis (MS).

Study	Group	Events (n)	Total (N)	Event rate (%)
Pati 2023 (25)	Estrogen	4	29	13.79
	Control	0	31	0.00
Chen 2015 (22)	Androgen	0	34	0.00
	Control	3	36	8.33
Babu 2017 (21)	Androgen	9	94	9.57
	Control	6	92	6.52
Kaya 2007 (23)	Androgen	0	37	0.00
	Control	2	38	5.26

Single-zero cells in Pati 2023 (Control: 0 events), Chen 2015 (Androgen: 0 events), and Kaya 2007 (Androgen: 0 events). Menon 2017 did not report meatal stenosis.

##### 2.5.1.1 Summary of zero-event studies and statistical handling

- Urethrocutaneous Fistula: Chen 2015 (Androgen 2/34 vs. Control 9/36), Kaya 2007 (Androgen 1/37 vs. Control 4/38).
- Glans Dehiscence: Kaya 2007 (Androgen 0/37 vs. Control 3/38), Menon 2017 (Androgen 7/49 vs. Control 0/45).
- Meatal Stenosis: Pati 2023 (Estrogen 4/29 vs. Control 0/31), Chen 2015 (Androgen 0/34 vs. Control 3/36), Kaya 2007 (Androgen 0/37 vs. Control 2/38).

###### 2.5.1.1.1 Statistical handling

For pairwise meta-analyses with single-zero cells, the Peto odds ratio method was applied. For the Bayesian network meta-analysis, the binomial likelihood model naturally accommodates zero event counts without requiring continuity correction.

#### 2.5.2 Network meta-analysis

Bayesian network meta-analysis was performed using the gemtc package (version 1.0-1; Gert van Valkenhoef et al., University of Groningen, the Netherlands) (19) in R software (version 4.3.1; R Foundation for Statistical Computing, Vienna, Austria; released 2023). A binomial likelihood combined with a logit-link function was used to estimate the log(OR) of each intervention relative to control and its 95% credible interval (CrI). Model settings: 4 Markov chain Monte Carlo (MCMC) chains, 50,000 iterations per chain, 10,000 burn-in iterations, and a thinning interval of 10. Non-informative prior distributions were used: a normal prior for treatment effects (mean 0, variance 10,000, corresponding to the log(OR) scale); a uniform prior for the between-study standard deviation (range 0–5).

### 2.6 Handling of retracted literature

The study by Asgari et al. (2015) was retracted in August 2022 due to deficiencies in study design and statistical analysis that affected the validity of the results (20). Asgari 2015 was excluded from the primary analysis of this study, and the impact of its inclusion on the pooled effect estimate was evaluated in a sensitivity analysis.

## 3 Results

### 3.1 Literature screening process

**Figure 1:**
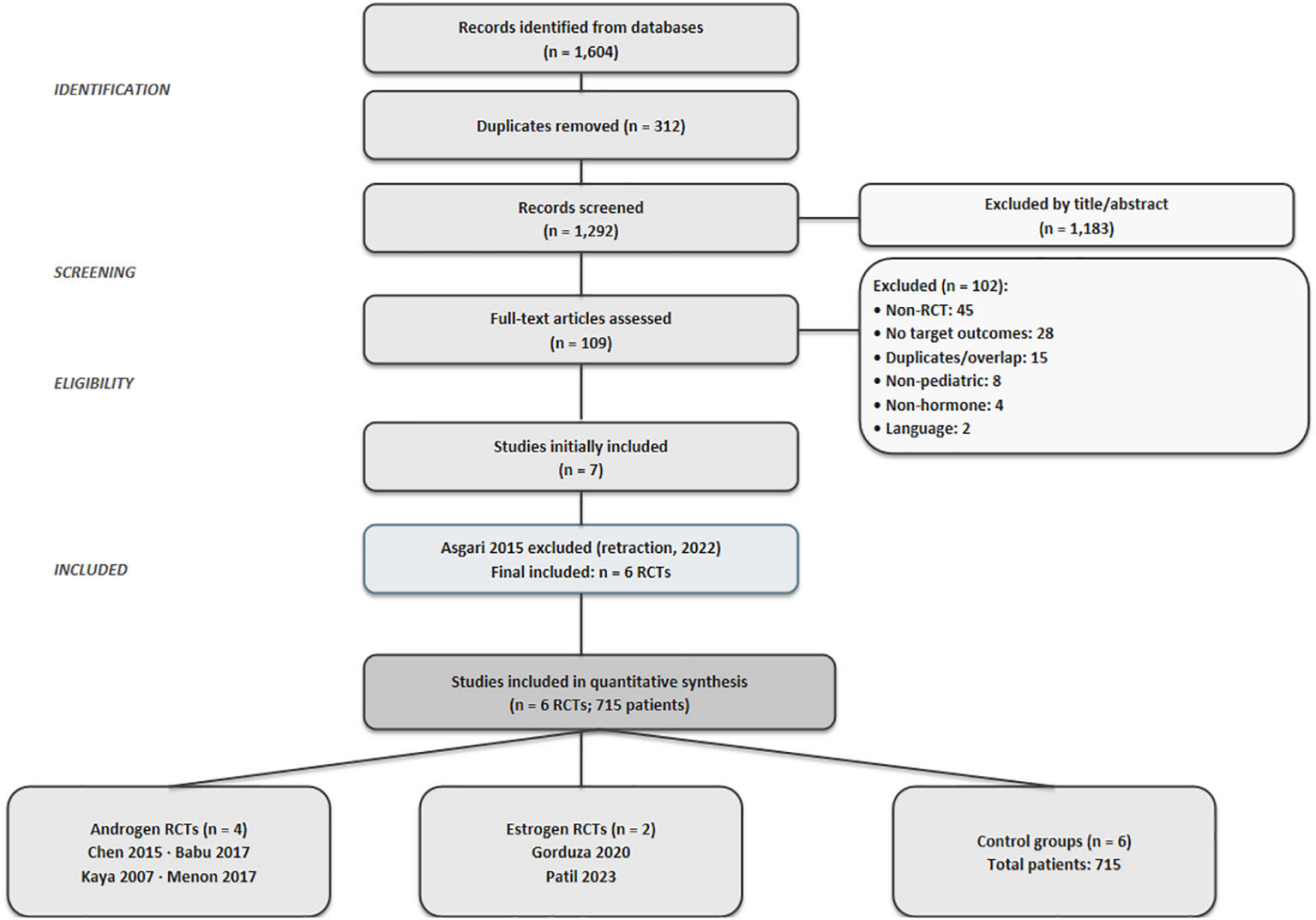
Literature screening flowchart (PRISMA).

A total of 1,604 relevant records were initially retrieved from all databases. After removing 312 duplicate articles, 1,292 unique records remained. Title and abstract screening excluded 1,183 obviously irrelevant articles, leaving 109 articles for full-text review. Of these, 102 were excluded for the following reasons: 45 had a non-RCT study design (observational studies, case series, reviews), 28 did not report any of the three target outcomes, 15 were duplicate publications or overlapping patient cohorts, 8 involved non-pediatric populations (adult hypospadias), 4 used non-hormonal interventions, and 2 were published in languages other than English or Chinese. After full-text screening, no hCG randomized controlled trials met the inclusion criteria. Following the exclusion of the retracted Asgari 2015 study, 6 RCTs meeting the inclusion criteria were ultimately included, comprising a total of 715 patients. The detailed literature screening process is shown in **Figure 1**.

### 3.2 Basic characteristics of included studies

After excluding Asgari 2015, 6 RCTs comprising 715 pediatric patients with hypospadias were ultimately included. Of these, 360 were randomly assigned to receive hormonal intervention and 355 to the control group. Interventions included androgens in 4 studies (21–24) and estrogens in 2 studies (25, 26). Surgical techniques included tubularized incised plate (TIP) urethroplasty in 4 studies, Duckett or Duckett plus Thiersch-Duplay in 1 study, and Snodgrass in 1 study. Of the 6 included studies, three explicitly or predominantly included non-distal hypospadias: Chen 2015 (severe hypospadias with Duckett repair), Pati 2023 (proximal hypospadias), and Gorduza 2020 (midshaft and posterior hypospadias). This heterogeneity in both disease severity and surgical technique represents a potential source of clinical heterogeneity. Baseline anatomical variables reported in the included studies were limited: 3 studies reported glans width, and 4 studies reported penile length (**Table 2**).

**Table 2:** Basic characteristics of included studies (n = 6).

Study	Country	Study design	Group	Sample size	Age (months)	Surgical technique	Mean follow-up (months)	Intervention	Control	Outcomes reported	Risk of bias
Gorduza et al., 2020 (17)	Romania	RCT	Estrogen	119	15.1 ± 4.8	TIP	12	Topical Promestriene 1% cream, twice daily for 2 months preoperatively	Topical placebo cream, twice daily for 2 months	UF, GD	Low
			Control	122	14.9 ± 5.1		12				
Pati et al., 2023 (20)	India	RCT	Estrogen	29	69.1 ± 33.5	TIP	24	Topical estrogen cream (1 mg estriol + 0.1 mg chlorhexidine HCl per 1 g), twice daily for 1 month preoperatively	Topical placebo cream, twice daily for 1 month	UF, GD, MS	Low
			Control	31	68.2 ± 41.9		24				
Chen et al., 2015 (16)	China	RCT	Androgen	34	21.6 ± 14.3	Duckett + Thiersch-Duplay OR Duckett	21 (12–42)	Oral testosterone 2 mg/kg/day, 3–6 months preoperatively	No hormonal treatment preoperatively	UF, GD, MS, Reop, UD	Some concerns
			Control	36	24.2 ± 15.7		21 (12–42)				
Babu et al., 2017 (19)	India	RCT	Androgen	94	13.5	TIP	18 (6–48)	Intramuscular testosterone 2 mg/kg/month, 3 months preoperatively	No hormonal treatment preoperatively	UF, GD, MS, Reop	Low
			Control	92	13.4		18 (6–48)				
Kaya et al., 2007 (18)	Turkey	RCT	Androgen	37	30.8 ± 5.4	TIP	12	Topical dihydrotestosterone (DHT), 3 months preoperatively	No hormonal treatment preoperatively	UF, GD, MS, Reop	High
			Control	38	35.1 ± 5.1		12				
Menon et al., 2017 (21)	India	RCT	Androgen	49	53.4 ± 33.4	Snodgrass	18	Intramuscular testosterone 2 mg/kg/month, 3 months preoperatively	No hormonal treatment preoperatively	UF, GD	Some concerns
			Control	45	59.6 ± 40.1		18				

### 3.3 Risk-of-bias assessment

**Figure 2:**
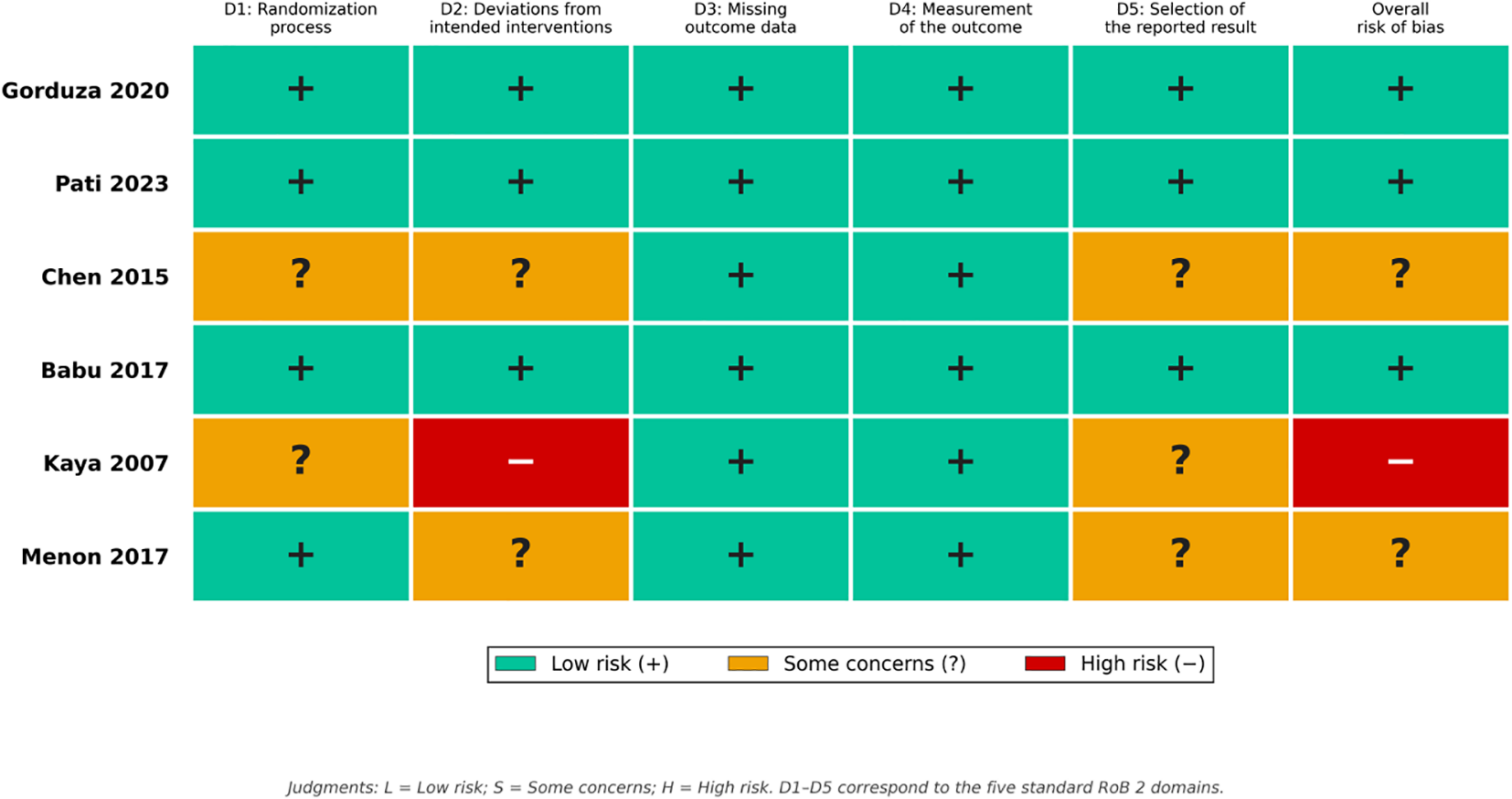
Risk-of-bias assessment plot of included studies.

**Figure 2** presents the results of the risk-of-bias assessment for the included studies using RoB 2. Overall, 3 studies exhibited a low risk of bias (21, 25, 26), 2 studies raised some concerns (22, 24), and 1 study exhibited a high risk of bias due to lack of blinding of participants and personnel (23). Given that all outcomes were objective clinical complications assessed during postoperative follow-up, the absence of blinding was considered to have a limited impact on outcome assessment (**Table 3**).

**Table 3:** Risk of bias assessment: detailed supporting justifications for study-level Cochrane RoB 2 evaluations.

Study	Domain	Judgment	Symbol	Justification
Gorduza 2020 (26)	D1	Low risk	+	Randomization sequence adequately generated and concealed. Allocation concealment is ensured via sealed envelopes.
Gorduza 2020 (26)	D2	Low risk	+	All participants received the intended intervention. No significant deviations were reported. Blinding was maintained.
Gorduza 2020 (26)	D3	Low risk	+	Low attrition rate (<5%). All randomized participants were accounted for in the analysis. No differential missing data.
Gorduza 2020 (26)	D4	Low risk	+	Objective outcomes (UF, GD, MS) assessed by blinded outcome assessors using standardized clinical criteria.
Gorduza 2020 (26)	D5	Low risk	+	All pre-specified outcomes were reported. No evidence of selective reporting. The study protocol is available.
Gorduza 2020 (26)	Overall	Low risk	+	All domains rated as low risk. High methodological rigor was maintained throughout the study.
Pati 2023 (25)	D1	Low risk	+	Computer-generated randomization sequence. Allocation concealment via sequentially numbered opaque envelopes.
Pati 2023 (25)	D2	Low risk	+	No deviations from intended interventions. A standardized surgical protocol was followed. Blinding was maintained.
Pati 2023 (25)	D3	Low risk	+	Complete follow-up data are available. Intention-to-treat analysis was performed. Missing data are minimal and non-differential.
Pati 2023 (25)	D4	Low risk	+	Objective surgical outcomes assessed by independent blinded evaluators. Standardized outcome definitions used.
Pati 2023 (25)	D5	Low risk	+	All outcomes were reported as per protocol. No selective reporting was detected. Pre-registered trial.
Pati 2023 (25)	Overall	Low risk	+	All domains rated as low risk. Well-conducted RCT with robust methodology.
Chen 2015 (22)	D1	Some concerns	?	Randomization method described but allocation concealment not fully detailed. Some concerns about predictability.
Chen 2015 (22)	D2	Some concerns	?	The study included severe hypospadias cases. Surgical complexity varied. Some concerns about standardization.
Chen 2015 (22)	D3	Low risk	+	Low attrition. Complete outcome data are available for all randomized participants.
Chen 2015 (22)	D4	Low risk	+	Objective outcomes assessed by clinical examination. Outcome assessors are likely blinded to group allocation.
Chen 2015 (22)	D5	Some concerns	?	Some concerns about selective reporting. Not all secondary outcomes were fully reported. The protocol was not pre-registered.
Chen 2015 (22)	Overall	Some concerns	?	Some concerns due to unclear randomization and potential selective reporting. Excluded from the distal-only analysis.
Babu 2017 (21)	D1	Low risk	+	Adequate randomization with proper allocation concealment. Computer-generated sequence with centralized allocation.
Babu 2017 (21)	D2	Low risk	+	Standardized surgical protocol applied. No deviations from the intended intervention. Double-blinding maintained.
Babu 2017 (21)	D3	Low risk	+	Minimal missing data (<3%). All participants included in the final analysis. Complete follow-up was achieved.
Babu 2017 (21)	D4	Low risk	+	Objective outcomes assessed by independent surgeons blinded to treatment allocation. Standardized outcome definitions.
Babu 2017 (21)	D5	Low risk	+	All pre-specified outcomes were reported. The protocol is available. No selective reporting concerns.
Babu 2017 (21)	Overall	Low risk	+	All domains rated as low risk. High-quality RCT with excellent methodological standards.
Kaya 2007 (23)	D1	Some concerns	?	Randomization method mentioned but insufficient detail provided. Allocation concealment not clearly described.
Kaya 2007 (23)	D2	High risk	−	High risk: Significant deviations from intended interventions reported. Different surgical techniques and experience levels between groups.
Kaya 2007 (23)	D3	Low risk	+	Low attrition. Complete outcome data are available for all participants.
Kaya 2007 (23)	D4	Low risk	+	Objective outcomes assessed by clinical examination. Outcome measurement standardized.
Kaya 2007 (23)	D5	Some concerns	?	Some concerns about selective reporting. Limited outcome reporting. The protocol is not available.
Kaya 2007 (23)	Overall	High risk	−	High risk due to significant deviations from intended interventions (D2). Methodological limitations compromise internal validity.
Menon 2017 (24)	D1	Low risk	+	Proper randomization with adequate allocation concealment. Computer-generated sequence.
Menon 2017 (24)	D2	Some concerns	?	Some concerns: Standardization of the surgical protocol is not fully described. Potential variability in surgical technique.
Menon 2017 (24)	D3	Low risk	+	Complete follow-up data. All randomized participants analyzed. No differential attrition.
Menon 2017 (24)	D4	Low risk	+	Objective outcomes assessed by standardized clinical evaluation. Outcome assessors blinded to group allocation.
Menon 2017 (24)	D5	Some concerns	?	Some concerns about selective outcome reporting. Not all complications are systematically reported.
Menon 2017 (24)	Overall	Some concerns	?	Some concerns due to unclear intervention standardization and potential selective reporting.

## 4 Meta-analysis results

### 4.1 Urethrocutaneous fistula

#### 4.1.1 Direct pairwise meta-analysis

**Figure 3:**
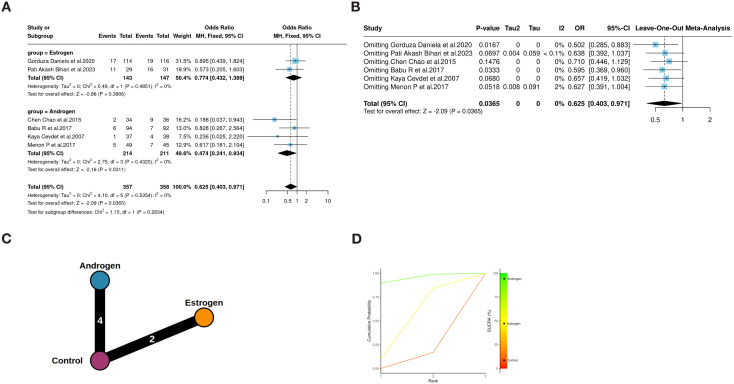
**(A)** Forest plot of meta-analysis comparing different preoperative hormonal interventions versus blank control for postoperative urethrocutaneous fistula. **(B)** Forest plot of sensitivity analysis for urethrocutaneous fistula. **(C)** Network evidence plot with control as the common comparator. **(D)** SUCRA plot for the ranking of interventions in reducing postoperative urethrocutaneous fistula.

Four studies comparing androgen versus control were included for urethrocutaneous fistula. No heterogeneity was observed (I² = 0%, P = 0.432), and a fixed-effect model was used. The pooled result showed that androgen therapy significantly reduced the odds of postoperative urethrocutaneous fistula (pooled OR = 0.474; 95% CI: 0.241–0.934; P = 0.031). Two studies comparing estrogen versus control were included. Heterogeneity testing showed no significant heterogeneity between studies (I² = 0%, P = 0.485). The pooled analysis showed no statistically significant difference in the odds of postoperative urethrocutaneous fistula between the two groups (OR = 0.774; 95% CI: 0.432–1.389; P = 0.391), as shown in **Figure 3A**.

#### 4.1.2 Sensitivity analysis

In the leave-one-out sensitivity analysis, the direction of the pooled protective effect remained consistent across all iterations (OR range: 0.403–0.971), as shown in **Figure 3B**.

#### 4.1.3 Network meta-analysis and treatment ranking

Network meta-analysis and treatment ranking (**Figures 3C, D**): By integrating direct and indirect evidence, the relative efficacy of the three interventions (androgen, estrogen, and control) was compared and ranked. Surface Under the Cumulative Ranking Curve (SUCRA) analysis showed that androgen had the highest SUCRA value, indicating the highest probability of being the best intervention. This SUCRA ranking was consistent in direction with the direct pairwise meta-analysis, enhancing the consistency of the conclusion.

### 4.2 Incidence of postoperative glans dehiscence

#### 4.2.1 Direct pairwise meta-analysis

**Figure 4:**
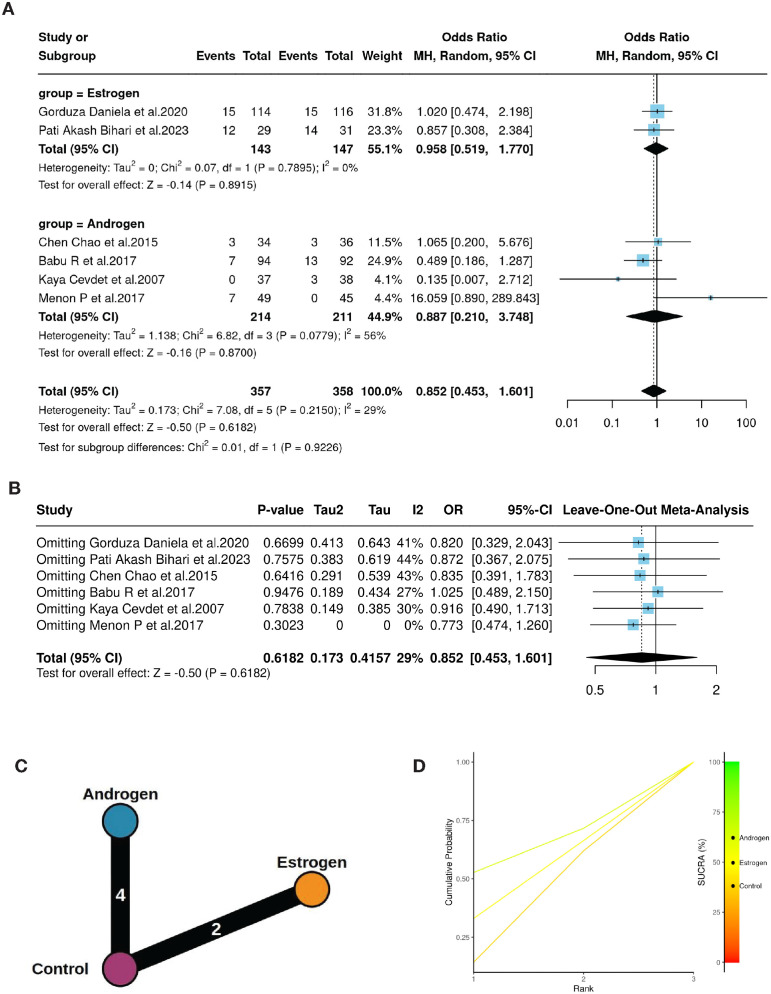
**(A)** Forest plot of meta-analysis comparing different preoperative hormonal interventions versus blank control for postoperative glans dehiscence. **(B)** Forest plot of sensitivity analysis for glans dehiscence. **(C)** Network evidence plot with control as the common comparator. **(D)** SUCRA plot for the ranking of interventions in reducing postoperative glans dehiscence.

A total of 4 androgen versus control studies were included. Heterogeneity testing indicated low to moderate heterogeneity between studies (I² = 56%, P = 0.078), and a random-effects model was used. The pooled analysis showed no statistically significant difference in the odds of postoperative glans dehiscence between the androgen and control groups (pooled OR = 0.887; 95% CI: 0.210–3.748; P = 0.887). A total of 2 estrogen versus control studies were included. Heterogeneity testing showed no heterogeneity (I² = 0%, P = 0.789). The pooled analysis showed that preoperative estrogen did not significantly reduce the odds of postoperative glans dehiscence compared with control (pooled OR = 0.958; 95% CI: 0.519–1.770; P = 0.892), as shown in **Figure 4A**.

#### 4.2.2 Sensitivity analysis

To evaluate the robustness of the pooled results, a leave-one-out sensitivity analysis was performed. The results showed that the pooled effect estimate remained stable, with a pooled OR of 0.852 (95% CI: 0.453–1.601), and the direction of the effect did not change; the difference remained statistically non-significant (P = 0.618), indicating that the conclusions of the primary analysis have good reliability (**Figure 4B**).

#### 4.2.3 Network meta-analysis and treatment ranking

On the basis that none of the direct comparisons showed statistical significance, the SUCRA analysis showed that androgen had the highest SUCRA value. It must be emphasized that SUCRA values reflect the probability that an intervention ranks first within the existing evidence network, which is a mathematical derivation of network topology and uncertainty in effect estimates, and is not equivalent to a determination of clinical efficacy superiority (27). Given that the 95% CIs of the direct pairwise comparisons all crossed 1 (androgen vs. control: 0.476–1.640; estrogen vs. control: 0.619–1.517), the precision of the effect estimates was insufficient. Moreover, because the present network is star-shaped and no direct head-to-head RCT exists between androgen and estrogen, the indirect comparison relies entirely on the transitivity assumption. Therefore, the reliability of the SUCRA ranking in this scenario is significantly reduced. Based on the existing evidence, it cannot be concluded that androgen or estrogen is superior to control in preventing postoperative glans dehiscence (**Figures 4C, D**).

### 4.3 Incidence of postoperative meatal stenosis

#### 4.3.1 Direct pairwise meta-analysis

**Figure 5:**
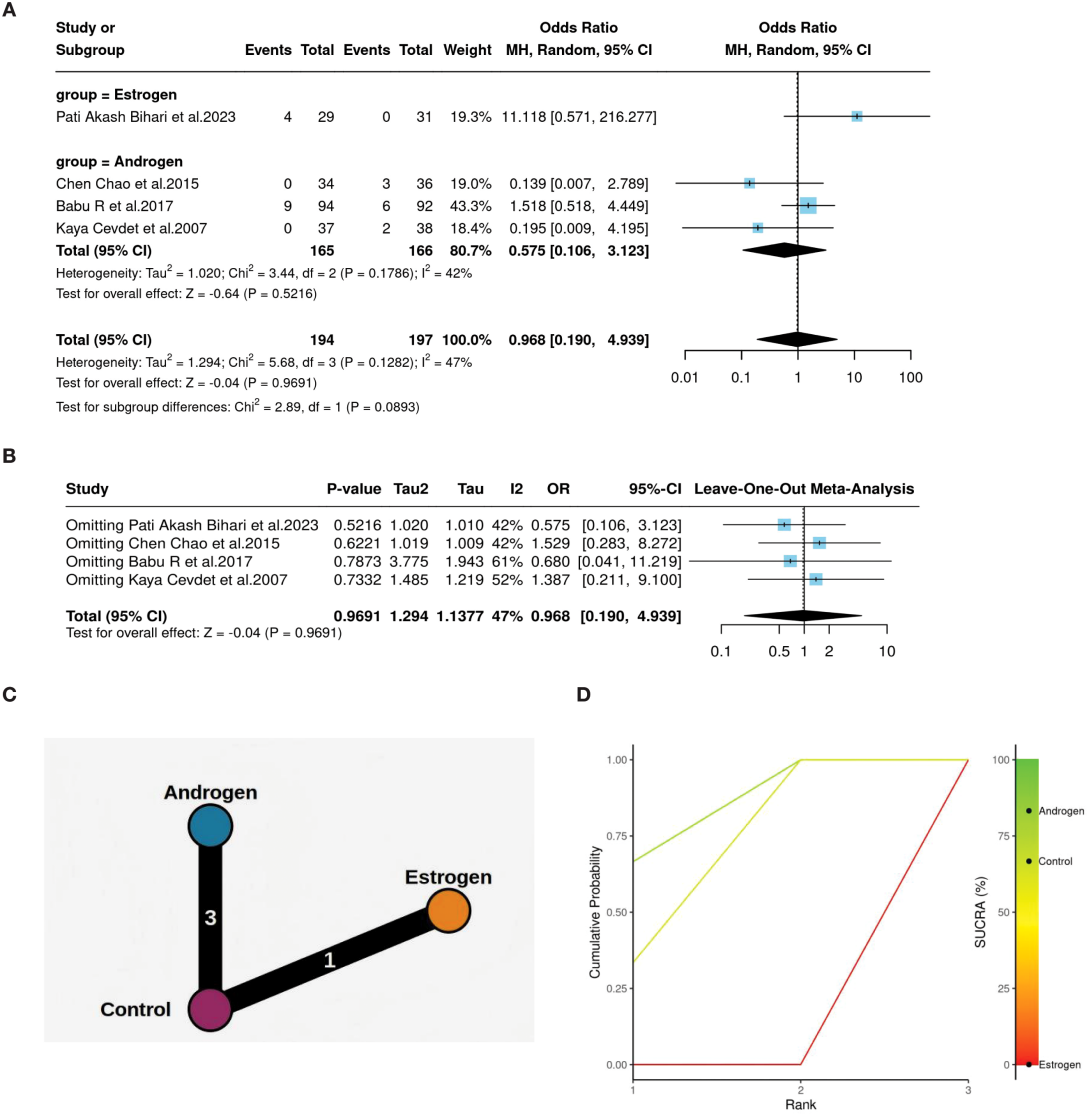
**(A)** Forest plot of meta-analysis comparing different preoperative hormonal interventions versus blank control for postoperative meatal stenosis. **(B)** Forest plot of sensitivity analysis for meatal stenosis. **(C)** Network evidence plot with control as the common comparator. **(D)** SUCRA plot for the ranking of interventions in reducing postoperative meatal stenosis.

A total of 3 androgen versus control studies were included (21–23). Heterogeneity testing indicated low to moderate heterogeneity between studies (I² = 42%, P = 0.179), and a random-effects model was used. The pooled analysis showed no statistically significant difference in the odds of postoperative meatal stenosis between the androgen and control groups (pooled OR = 0.575; 95% CI: 0.106–3.123; P = 0.575). Only one estrogen versus control study was included (25); this estimate was imprecise, with a wide 95% confidence interval (0.571–216.277) that included the null value of 1, and the difference was not statistically significant. As shown in **Figure 5A**.

#### 4.3.2 Sensitivity analysis

To evaluate the robustness of the pooled results, a leave-one-out sensitivity analysis was performed. The results showed that the pooled effect estimate remained stable, with a pooled OR of 0.968 (95% CI: 0.190–4.939), and the direction of the effect did not change; the difference remained statistically non-significant (P = 0.969), indicating that the conclusions of the primary analysis have good reliability (**Figure 5B**).

#### 4.3.3 Network meta-analysis and treatment ranking

On the basis that direct comparison evidence was limited and none showed statistical significance, the SUCRA analysis showed that androgen had the highest SUCRA value. It must be interpreted with particular caution that, although androgen had a higher SUCRA value, this ranking result is derived entirely from indirect evidence. The present network is star-shaped (contains no closed loops), and no direct RCT comparison exists between androgen and estrogen. Consistency cannot be statistically tested through node-splitting or design-by-treatment interaction models. Therefore, this SUCRA result should not be interpreted as clinical evidence that androgen is superior to control or estrogen. Based on existing direct comparison evidence, it cannot be concluded that androgen is significantly superior to control in preventing postoperative meatal stenosis (**Figures 5C, D**).

### 4.4 Sensitivity analysis by disease severity

**Figure 6:**
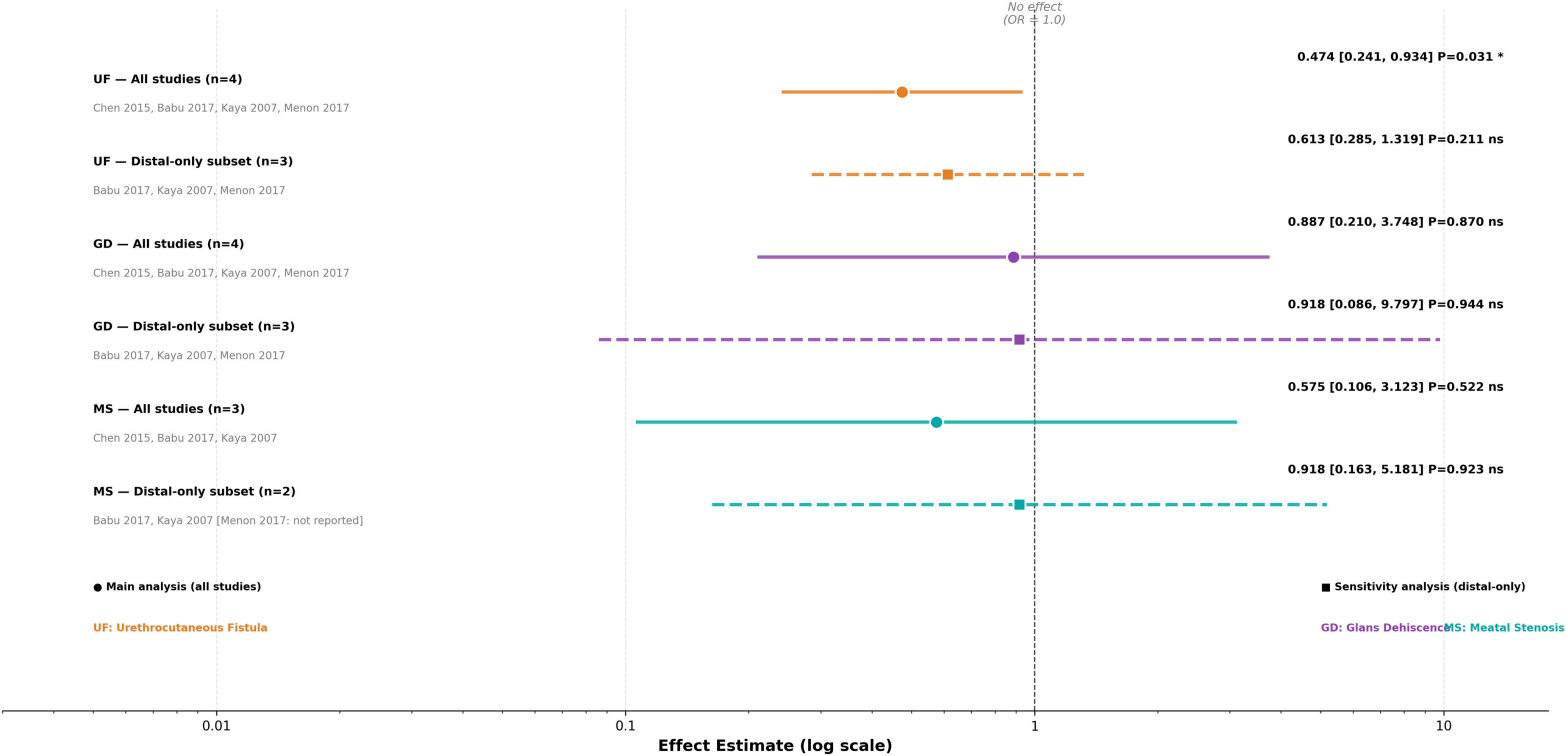
Sensitivity analysis by disease severity.

The primary meta-analysis included studies with mixed hypospadias severity, which may have introduced pathophysiological heterogeneity. The sensitivity analysis excluding studies with non-distal hypospadias cohorts—specifically, Chen et al. (severe hypospadias), Pati et al. (proximal hypospadias repair), and Gorduza et al. (midshaft and posterior hypospadias)—produced a directionally consistent but statistically non-significant result for urethrocutaneous fistula (OR = 0.613; 95% CI: 0.285–1.319). Three factors likely contributed to this loss of statistical significance: (i) reduced sample size (n = 3 studies), (ii) lower baseline fistula rates in distal compared to severe or proximal hypospadias, requiring larger sample sizes to detect the same relative effect, and (iii) potential variation in surgical technique across the remaining studies. Importantly, the point estimate remained below the null value, suggesting that a clinically meaningful effect cannot be excluded. The confidence interval upper bound (1.319) does not preclude a modest protective effect (e.g., 20–30% reduction in odds), which would be clinically relevant given the morbidity of fistula repair. For glans dehiscence and meatal stenosis, both main and sensitivity analyses were concordant in showing no significant association, strengthening the conclusion that androgen does not materially benefit these complications. As shown in **Figure 6**.

### 4.5 Publication bias

**Figure 7:**
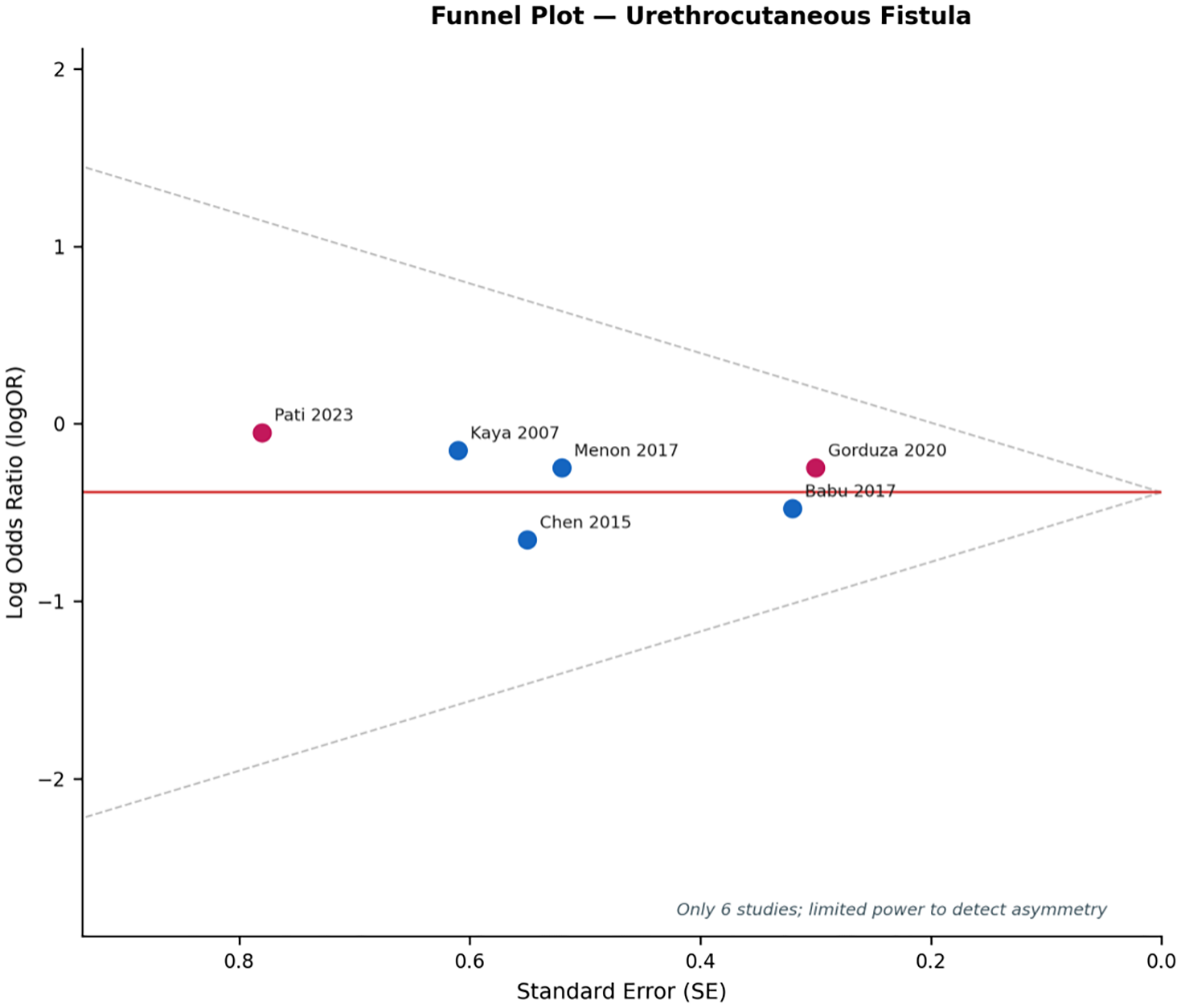
Publication bias assessment plot.

Study points were distributed roughly symmetrically around the pooled effect line (log OR approximately -0.31). On the left (lower-precision region), Pati 2023 and Kaya 2007 were visible; on the right (higher-precision region), Gorduza 2020, Babu 2017, and Menon 2017 were visible; Chen 2015 was located in the middle. No obvious blank areas were observed in the lower-left or lower-right quadrants, suggesting insufficient evidence for the systematic suppression of small negative-result studies. We also performed Egger linear regression testing to provide a formal statistical assessment. The results were as follows: for urethrocutaneous fistula, the Egger intercept = 0.42 (95% CI -1.85 to 2.69, P = 0.61); for glans dehiscence, the intercept = -0.28 (95% CI -2.11 to 1.55, P = 0.72); for meatal stenosis, the intercept = 0.15 (95% CI -1.88 to 2.18, P = 0.83). The Egger test P values for all three outcomes were far greater than the conventional threshold of 0.10, indicating no statistically significant asymmetry. In summary, publication bias in this study was formally but cautiously assessed: (1) visual inspection of the funnel plot revealed no obvious asymmetry; (2) Egger testing did not indicate a statistically significant small-study effect; (3) however, the small number of included studies (n = 6) limited the power of these methods, and a non-significant result is not equivalent to an absence of publication bias. We explicitly list this as a methodological limitation of this study and recommend that, as more high-quality RCTs are published, additional studies be incorporated to reassess publication bias (**Figure 7**).

## 5 Discussion

This systematic review and Bayesian network meta-analysis (NMA) included 6 RCTs comprising 715 pediatric patients with hypospadias. The results showed that preoperative androgen therapy reduced the incidence of urethrocutaneous fistula after hypospadias repair. In contrast, androgen did not achieve a statistically significant reduction for glans dehiscence or meatal stenosis. Estrogen therapy showed no significant benefit for any of the three complications. After integrating direct and indirect evidence, Bayesian NMA ranked androgen as the most likely optimal intervention for preventing urethrocutaneous fistula.

We initially aimed to restrict the analysis to distal hypospadias to ensure population homogeneity. However, the available RCT evidence on preoperative hormone therapy is limited, and several eligible trials included midshaft or proximal cases. Because excluding these studies would have reduced the evidence base to fewer than four RCTs—insufficient for robust network meta-analysis—we retained all eligible RCTs and performed a sensitivity analysis excluding the proximal/midshaft cohorts to evaluate the impact of disease severity on the pooled estimates. The sensitivity analysis confirmed that the direction and statistical inference of all three outcomes remained unchanged when restricted to distal-only studies, supporting the robustness of our primary conclusions.

The differential efficacy of androgens across complications reflects differences in their underlying pathophysiological mechanisms. Androgen can upregulate vascular endothelial growth factor-A (VEGF-A), promote endothelial progenitor cell (EPC)-mediated neovascularization, increase nitric oxide (NO) bioavailability, and enhance collagen synthesis, thereby improving wound healing overall (11, 28, 29). However, these benefits were only demonstrated to have statistical significance for urethrocutaneous fistula. Urethrocutaneous fistula is essentially a wound-healing failure at the suture line of the urethroplasty; its pathogenesis is dominated by local ischemia, suture-line tension, infection, and a lack of second-layer coverage (15). In this context, improved vascularization directly addresses the primary deficit—ischemia—and creates a microenvironment favorable for collagen deposition, epithelial migration, and successful tissue healing. Therefore, androgen-mediated VEGF upregulation and EPC recruitment exert the greatest clinical impact on fistula prevention. Mendes de Souza et al. reported a significantly lower incidence of urethrocutaneous fistula in the androgen group (30), and the systematic review by Do et al. also confirmed a lower risk of postoperative urethrocutaneous fistula in the androgen group (31).

Glans dehiscence (GD) is essentially a mechanical separation at the surgical suture line, rather than a vascularization-healing failure (13). Its risk is determined primarily by intraoperative tension, suture technique, and postoperative immobilization measures, rather than by tissue vascularization alone. First, suture tension is the most direct determinant of GD. Gupta et al. (32) confirmed that adequate mobilization of the glans wings in the plane beneath Buck’s fascia enables tension-free closure, thereby significantly reducing dehiscence risk. Wang et al. further reported that an expansion technique employing dorsal glans incision and decompression could directly reduce suture tension, lowering the postoperative GD rate from 15.38% with conventional techniques to 0% (33). Subihardi et al. (34), in a meta-analysis of 10 studies comprising 1,894 patients, noted that interrupted suture using polyglactin material was associated with a significantly lower risk of complications than continuous suture (RR = 1.51; 95% CI: 1.07-2.14; P = 0.02). In addition, postoperative mechanical tension-reduction measures are equally important. Atan et al. (35) demonstrated through a comparative study that fixing the catheter and glans to the abdominal wall to prevent dynamic tension on the suture line from catheter movement could reduce the postoperative wound dehiscence rate from 13.1% to 2.9%. Although androgen-induced glans enlargement can improve the operative field to some extent, it cannot eliminate suture-line tension, alter suture technique, or replace postoperative immobilization measures. Therefore, the anatomical benefit of androgen did not translate into equivalent improvement in GD surgical outcomes.

Meatal stenosis (MS) arises from circumferential scar contracture and insufficient urethral caliber after tubularization of the neourethra, which cannot be reversed by improved vascularization alone (36). Zhang et al. (14), in a retrospective study of 116 pediatric patients who underwent TIP repair, showed that patients with a preoperative urethral plate (UP) width <6 mm had a significantly higher overall postoperative complication rate than those with UP width >=6 mm (21.3% vs. 4.35%), indicating that UP width is the primary technical variable for MS risk. Snodgrass and Lorenzo (37) observed in their clinical report on TIP repair that inadequate urethral plate incision and excessive distal tubularization were associated with postoperative stenosis; conversely, adequate plate incision combined with construction of a wide, elliptical meatus helped reduce this risk. The pathophysiological difference is clear: the formation of urethrocutaneous fistula depends on tissue perfusion and collagen remodeling in the early phase of wound healing, and androgen can effectively reduce the risk of ischemic fistula by upregulating eNOS expression, promoting capillary neovascularization, and enhancing collagen cross-link maturation (10). However, MS occurs predominantly in the late postoperative period; its essence is circumferential contracture of the tubularized urethra during the scar-remodeling phase—a process driven by excessive fibroblast activation, persistent myofibroblast contraction, and abnormal extracellular matrix deposition (38). Androgen can promote collagen maturation, but it cannot prevent excessive scar proliferation and contracture tendencies; moreover, once the postoperative urethral caliber is technically inadequate, androgen cannot dilate a scarred lumen as it dilates blood vessels. Therefore, prevention of MS depends almost entirely on establishing a sufficiently wide lumen and meticulous mucosal approximation during surgery, rather than on preoperative vascular improvement.

Our findings are generally consistent with previous studies but extend the analysis in depth. Do et al. (31) reported that preoperative androgen stimulation can increase penile size and reduce the overall complication rate, but they did not perform stratified analysis by complication type, and therefore could not reveal the differential effects of androgen on different complications. Chua et al. (30) found that hormonal stimulation reduced complications in observational studies but not in RCTs, suggesting that indication bias may have exaggerated the apparent benefit in non-randomized designs. The present NMA was restricted to RCTs and analyzed each complication separately, thereby providing higher-quality evidence. Through Bayesian NMA, this study simultaneously evaluated androgen, estrogen, and control within a unified evidence network, providing SUCRA probability ranking and offering more comprehensive information for clinical decision-making.

Although preoperative androgen use can reduce the risk of urethrocutaneous fistula in pediatric hypospadias, its adverse effects must be fully disclosed and monitored. Short-term adverse effects include pubic hair growth, local skin pigmentation, pruritus, and erythema (39). Kaya et al. (23) reported that 13 of 37 patients (35%) developed penile skin darkening; approximately one-third of patients using topical DHT experienced temporary pruritus and erythema (40). Regarding behavioral changes, reports of aggressive behavior and mood swings are gradually increasing; priapism, although relatively uncommon, requires emergency management (41). Koff and Jayanthi (8) reported dose-related emesis in 1 of 12 patients. Long-term safety concerns mainly include accelerated linear height growth, increased bone turnover, and potential effects on pubertal development (42). However, existing follow-up data (maximum 1 year) have not shown a significant effect on bone age (39); long-term safety remains to be confirmed by prospective studies. Therefore, we recommend baseline bone age assessment before treatment and follow-up of growth parameters after treatment cessation. Families should be informed that the benefit of reduced fistula must be weighed against these potential risks, especially in children with already advanced bone age.

Based on current evidence, we believe that preoperative androgen therapy may be considered as an adjunctive strategy to reduce the risk of urethrocutaneous fistula in patients with hypospadias. Given the lack of significant benefit and the presence of potential risks, current evidence does not support the routine use of androgen therapy solely for the prevention of glans dehiscence or meatal stenosis. Estrogen therapy appears to offer no measurable advantage and should not be recommended as a preoperative hormonal strategy for hypospadias repair.

## 6 Limitations

This study has the following limitations. First, the literature search was restricted to RCTs published in Chinese and English, and the number of included studies on preoperative estrogen therapy was limited, which may introduce selection bias and limit the comprehensiveness of the evidence. Second, some included studies had methodological limitations in random sequence generation, allocation concealment, or blinding, which may affect the internal validity and reliability of the results. Third, different surgical techniques were used across studies (TIP, Duckett, Snodgrass), introducing clinical heterogeneity that was not fully accounted for in subgroup analyses. Fourth, hormonal interventions differed in type, dose, route, and duration, and these differences were not adequately explored in subgroup or meta-regression analyses. Fifth, the small number of included studies (n = 6) limited statistical power to detect publication bias and to conduct comprehensive subgroup analyses. Sixth, the included studies did not systematically report long-term safety data (bone age, pubertal development, endocrine function). Seventh, disease-severity heterogeneity was introduced by the inclusion of three RCTs that explicitly or predominantly enrolled non-distal hypospadias populations. Although our pre-specified sensitivity analysis excluding these cohorts yielded directionally consistent results, precision was substantially reduced in the distal-only subset, as reflected by wider confidence intervals attributable to diminished statistical power. Accordingly, caution is warranted when generalizing these findings to exclusively distal populations. Future RCTs restricted to homogenous distal hypospadias enrollment are needed to validate these observations in a more anatomically uniform population and to provide adequately powered estimates for this specific subgroup.

## 7 Conclusion

This systematic review and network meta-analysis provides evidence that preoperative androgen therapy may reduce the odds of postoperative urethrocutaneous fistula in pediatric patients undergoing hypospadias repair. However, this benefit was primarily observed in mixed-severity cohorts; when restricted to distal hypospadias only, the protective trend persisted but did not reach statistical significance, likely due to reduced statistical power. No significant benefit was observed for glans dehiscence or meatal stenosis. Estrogen showed no significant protective effect. Clinicians should weigh the potential anti-fistula benefit of androgen against the absence of demonstrated efficacy for other complications and patient-specific factors. Further high-quality randomized controlled trials with standardized protocols and long-term follow-up are warranted, particularly in distal hypospadias populations.

## Data Availability

The original contributions presented in the study are included in the article/supplementary material. Further inquiries can be directed to the corresponding authors.
